# Genome-wide hydroxymethylation profiles in liver of female Nile tilapia with distinct growth performance

**DOI:** 10.1038/s41597-023-01996-5

**Published:** 2023-03-01

**Authors:** Ioannis Konstantinidis, Pål Sætrom, Jorge M. O. Fernandes

**Affiliations:** 1 Faculty of Biosciences and Aquaculture, Nord University, Bodø, Norway; 2 Department of Clinical and Molecular Medicine, Norwegian University of Science and Technology, NTNU, Trondheim, Norway; 3 Department of Computer Science, Norwegian University of Science and Technology, NTNU, Trondheim, Norway; 4 Bioinformatics core facility-BioCore, Norwegian University of Science and Technology, NTNU, Trondheim, Norway; 5 K.G. Jebsen Center for Genetic Epidemiology, Norwegian University of Science and Technology, NTNU, Trondheim, Norway

**Keywords:** Epigenetics, DNA methylation

## Abstract

The mechanisms underlying the fast genome evolution that occurs during animal domestication are poorly understood. Here, we present a genome-wide epigenetic dataset that quantifies DNA hydroxymethylation at single nucleotide resolution among full-sib Nile tilapia (*Oreochromis niloticus*) with distinct growth performance. In total, we obtained 355 million, 75 bp reads from 5 large- and 5 small-sized fish on an Illumina NextSeq500 platform. We identified several growth-related genes to be differentially hydroxymethylated, especially within gene bodies and promoters. Previously, we proposed that DNA hydroxymethylation greatly affects the earliest responses to adaptation and potentially drives genome evolution through its targeted enrichment and elevated nucleotide transversion rates. This dataset can be analysed in various contexts (e.g., epigenetics, evolution and growth) and compared to other epigenomic datasets in the future, namely DNA methylation and histone modifications. With forthcoming advancements in genome research, this hydroxymethylation dataset will also contribute to better understand the epigenetic regulation of key genomic features, such as cis-regulatory and transposable elements.

## Background & Summary

Animal domestication has been associated with early changes in gene expression^1^ and phenotypic divergence^2^, in addition to transcriptionally relevant genetic mutations that often require longer periods of time to emerge. Previous studies have shown that epigenetic modifications are likely involved in the process of adaptation under captivity^2,3^, overlap with genetic mutations after several generations and potentially drive genome evolution^4^. These recent findings have broadened our knowledge and understanding of how epigenetic modifications alter rapidly gene expression and contribute towards normal development and phenotypic divergence, including potentially pathological conditions such as diabetes^5^ and cancer^6^.

Growth is one of the main traits of interest in farmed animals intended for food production. One of the primary tissues involved in somatic growth is the liver, which is the main binding target of growth hormone and essential in insulin-growth factor I production. In teleosts, the liver is also an important organ for the synthesis of several yolk proteins (e.g., vitellogenin) which are transported to the oocytes for uptake^7^. Therefore, epigenetic modifications capable of altering hepatic gene expression in females have impact on egg size and quality, which are linked to the growth potential of the offspring^8^.

DNA 5-hydroxymethylcytosine (5hmC) is an oxidized derivative of 5-methylcytosine (5mC) that has received very little attention in the field of epigenetics despite its profound stability and association with critical molecular functions, namely gene transcription and histone accessibility^9–15^. It forms slowly right after DNA replication by ten-eleven translocation (TET) enzymes as opposed to 5mCs which are formed during DNA replication by DNA methyltransferases^16^. Although DNA hydroxymethylation has been characterized as a unique epigenetic modification with distinct roles in gene regulation^17^, methods such as reduced representation and whole genome bisulfite sequencing (RRBS and WGBS, respectively) produce data that make 5mCs and 5hmCs indistinguishable. To date, liver DNA hydroxymethylation and the role of TET enzymes have only been described in human diseases including hepatocellular carcinoma^18^ and non-alcoholic fatty liver disease (NAFLD)^19^. In our previous work, we have shown that DNA hydroxymethylation is an abundant epigenetic modification within the somatotropic axis, including the liver^20^. Interestingly, genes such as *ppargc1a*, which is associated with metabolic reprogramming and the coordination of glucose and fatty acid metabolism in the liver, are involved in the function of TET enzymes^21^.

Recently, we performed a genome-wide mapping of 5hmCs at single nucleotide resolution using reduced representation hydroxymethylation profiling (RRHP) and we captured 68% (1,096,820) of the total CCGG sites (1,613,446) across the Nile tilapia genome. Based on our data filtering methodology, we identified 138,000 significantly hydroxymethylated cytosines in two groups of Nile tilapia with distinct growth rates. Statistical analysis revealed 2,677 DhmCs (q < 0.05), out of which, 2,237 had significantly higher levels of DNA hydroxymethylation in large fish compared to their smaller counterparts (Supplementary file 1a-c; please see section “Data Records”).

Overall, we showed that DNA hydroxymethylation levels differ significantly in the liver of full-sib Nile tilapia with distinct growth rates during their early domestication. Our annotation revealed several differentially hydroxymethylated cytosines (DhmCs) that are particularly enriched within gene bodies and promoters, supporting their functional significance in transcriptional regulation. Furthermore, genes involved in growth and metabolic functions had higher DNA hydroxymethylation levels in large- than in small-sized fish (Supplementary file 2; please see section “Data Records”). Finally, we proposed that DNA hydroxymethylation is an epigenetic marker that likely regulates gene transcription and contributes to phenotypic divergence and adaptation in animals undergoing domestication. We are confident that this dataset has a great future potential because it addresses for the first time the changes of the DNA hydroxymethylome in phenotypically divergent full-sib animals during their early domestication. Forthcoming developments in genome annotation will likely provide valuable clues towards the understanding of how these epigenetic marks regulate gene expression, phenotypic divergence and genome evolution.

## Methods

A more detailed description of the experimental and computational methodology is described in our sister publication^2^. Briefly, wild Nile tilapia were captured from Nile River in Luxor, Egypt. As a mouthbrooding fish species, female Nile tilapia with eggs in their mouths were captured and their eggs were collected and transported to our breeding facility in Bodø, Norway. After a successful reproduction cycle using our founder population (F0), we observed phenotypic divergence among full-siblings in the first generation born in captivity (F1).

Sample size (n) was calculated using two methods. First, because RRHP produces a count-based data set similar to RNA-Seq, 5 individuals with a sequencing depth of approximately 20 million reads are able to distinguish 10-fold transcriptional difference^22^. Furthermore, RRHP using human samples can detect low 5hmC signals (20%, q < 0.05) with only 20–30 million reads^23^. In this study, the average depth among libraries was over 36 million reads, even if the Nile tilapia genome contains only 1.6 million CCGGs compared to 4.6 million in the human genome. Second, sample size was also calculated by taking into consideration the weight and length of the fish. Since the endpoint of this analysis was quantitative data (5hmC counts), we used the formula [n = 2·(SD·(Z_0_ + Z_≠_)/d)2], where SD stands for standard deviation among all individuals, Z_0_ from Z table and a = 0.05, Z_≠_ from Z table (power at 80%) and d-difference between mean weights which resulted in n = 4.04.

### Ethics approval

Animal handling and procedures were performed in accordance to the EU Directive 2010/63 on the use of animals for scientific purposes, and approved by the Norwegian Animal Research Authority (FOTS license no. 1042) and Nord University’s ethics committee.

### Sampling procedure

**Fig. 1:**
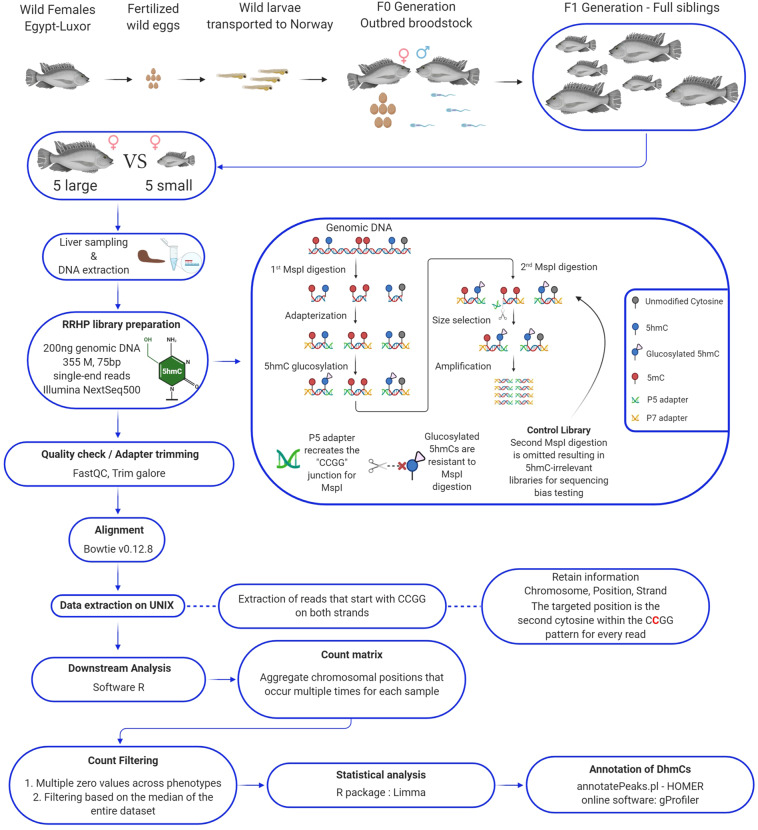
Flowchart of the experimental design and computational pipeline for downstream analysis of genome-wide hydroxymethylation by reduced representation hydroxymethylation profiling (RRHP).

In total, 10 full-sib Nile tilapia females (large- and small-size individuals, n = 5) were reared for 5 months in a 1500 L tank which was connected to the recirculating aquaculture system at Nord University’s research station (Fig. 1). All fish were caught by net and euthanized by clove oil overdose for 3 minutes at 28 °C. Pure clove oil (Sigma Aldrich, USA) was diluted in 1:10 (v/v) in 96% ethanol, and 20 ml of the mix was added for every 10 L of water in a 20 L oxygenated tank. Liver tissue was carefully dissected from the left lobe near the entry point of the portal vein. All samples were placed in 2 ml sterile cryogenic tubes, frozen instantly in liquid nitrogen and stored in −80 °C. The fish of this experiment represent our first generation (F1) of Nile tilapia born in captivity, as shown in Fig. 1. Detailed information on the animals used in this experiment can be found in Table 1.

**Table 1:** Morphometrics of Nile tilapia individuals at time of sampling (5 months old).

Tag	Sex	Tissue Sample	Size Code	Sample tag	Weight (g)	Total Length (cm)	Standard Length (cm)
F1-5mts-1	female	Liver	B	BL1	512	29	24
F1-5mts-6	female	Liver	B	BL2	634	30.2	25.5
F1-5mts-8	female	Liver	B	BL3	587	29.5	24.5
F1-5mts-11	female	Liver	B	BL4	584	28.5	23.5
F1-5mts-12	female	Liver	B	BL5	556	29	23.7
F1-5mts-3	female	Liver	S	SL1	191	21.4	17.5
F1-5mts-7	female	Liver	S	SL2	113	17.8	14
F1-5mts-9	female	Liver	S	SL3	138	18.6	15
F1-5mts-13	female	Liver	S	SL4	192	21.6	17.7
F1-5mts-14	female	Liver	S	SL5	129	19.3	15.7

### Extraction of DNA for RRHP

DNA was extracted using the Quick-DNA miniprep plus kit (Zymo Research, USA), following the protocol recommended for solid tissues without any modifications. Briefly, 10 mg of liver was added in a solution of 95 μl DNAse and RNAse free water, 95 μl solid tissue buffer and 10 μl Proteinase K (20 mg/ml). The mixture was vortexed and incubated for 3 hours at 55 °C. The tissue was solubilized in 400 μl of genomic binding buffer. DNA integrity was measured using TapeStation 2200 (Agilent Technologies, USA) and DNA purity was determined on a NanoDrop Spectrophotometer 1000 (ThermoFisher Scientific, USA). Prior to library preparation, DNA concentration and subsequent dilutions were measured using the dsDNA broad range assay on a Qubit 3.0 fluorometer (ThermoFisher Scientific).

### RRHP library preparation and sequencing

**Fig. 2:**
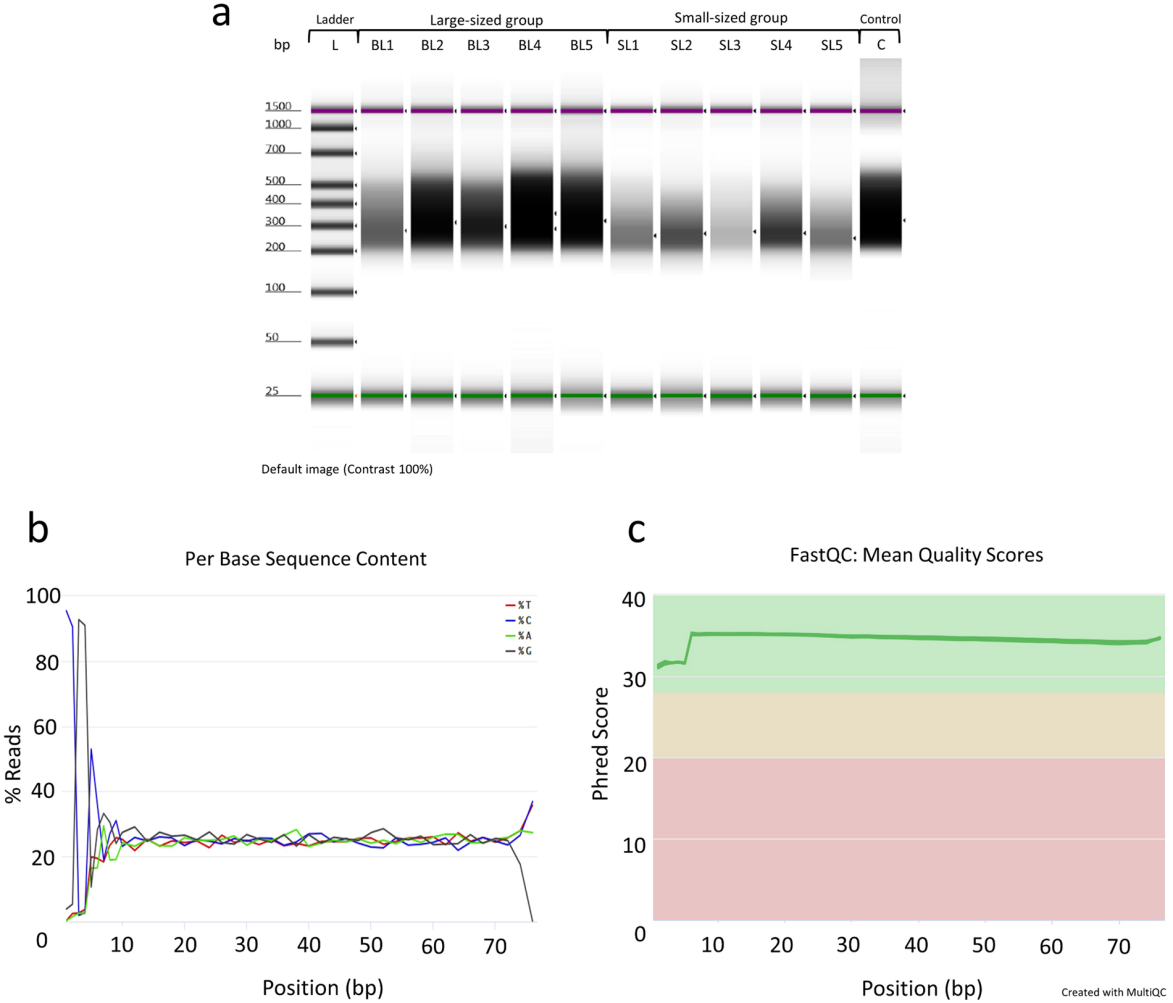
RRHP library integrity and quantification after size selection and purification using DNA high-sensitivity tape on a Tapestation 2200, and processing of sequenced reads. (**a**) From left to right, L is the DNA high-sensitivity ladder, BL1-BL5 represent the large-sized individuals, respectively. SL1-SL5 wells represent the small-sized individuals, respectively. C is the positive (5hmC-irrelevant) control library. Dark areas correspond to the size and concentration of the RRHP libraries after final purification. (**b**) A representative example of the per base sequence content for RRHP libraries during quality control using the FastQC program. (**c**) Visualization of the mean quality scores across all RRHP libraries using the MultiQC software.

Library construction for reduced representation 5hmC profiling was performed according to the manufacturer’s protocol (RRHP – Zymo Research) as described in our sister paper^2^. For the present dataset, we used 200 ng of genomic DNA from liver as input for the preparation of every library including a positive 5hmC-irrelevant control. Genomic DNA was digested for 8 hours at 37 °C using the restriction enzyme MspI (Fig. 1). The DNA fragments were adapter ligated and extended with RRHP-specific adapters. Here, the P5 adapter reconstitutes the restriction site CCGG at the junction while the P7 destroys it. After the glucosylation step, fragments with glucosylated 5hmC modifications at the CCGG junctions were resistant to the second MspI digestion, and thus retained their P5 adapters as opposed to fragments with unmodified or methylated cytosines which were cleaved. For our control library, the second MspI digestion was omitted resulting in the amplification of all adapterized fragments (unrelated to 5hmC content). All libraries were size selected and amplified for a total of 10 cycles according to the protocol. Samples with higher levels of DNA hydroxymethylation produce a higher number of adapter ligated fragments; therefore, the libraries were sequenced with equal volume rather than equal molarity. Library integrity was assessed using DNA high-sensitivity Tapestation 2200 (Fig. 2a). The libraries were sequenced using two high output, 75-cycle sequencing kits in single-end reading mode, using Nord University’s NextSeq500 Illumina platform. For the control libraries, half of the volume was sequenced in each flow cell to test for flow cell effects and save sequencing depth. After verifying that the two controls had no flow cell effects, the two controls were merged into a single fastq file. For both flow cells, final library pools were diluted to 1.5 nM prior to sequencing and 34% PhiX was included to account for the low complexity (CCGG) in the first 4 sequencing cycles.

### Processing and alignment of RRHP sequenced reads

Base call files were demultiplexed and converted to fastq files using the bcl2fastq v2.20.0.422 software (Illumina). Samples were identified based on their index sequence and concatenated accordingly in LINUX environment. Quality control of the sequenced reads was performed using the FastQC program^24^. The per base sequence content revealed the low complexity (CCGG) at the start of most reads which is typical for RRHP libraries (Fig. 2b). The mean quality scores of all samples were plotted using the MultiQC program^25^ (Fig. 2c). Adaptor sequences were removed using trim_galore v0.4.4^26^ (Supplementary File 3; please see section “Data Records”) and reads were aligned to the latest Nile tilapia genome^27^ using bowtie v0.12.8^28^ (Supplementary File 4; please see section “Data Records”) with the parameters–chunkmbs 1000 -S -v 1 -n 1 -m 3–strata–best -p 32.

### Extraction, filtering and differential analysis of RRHP data

**Fig. 3:**
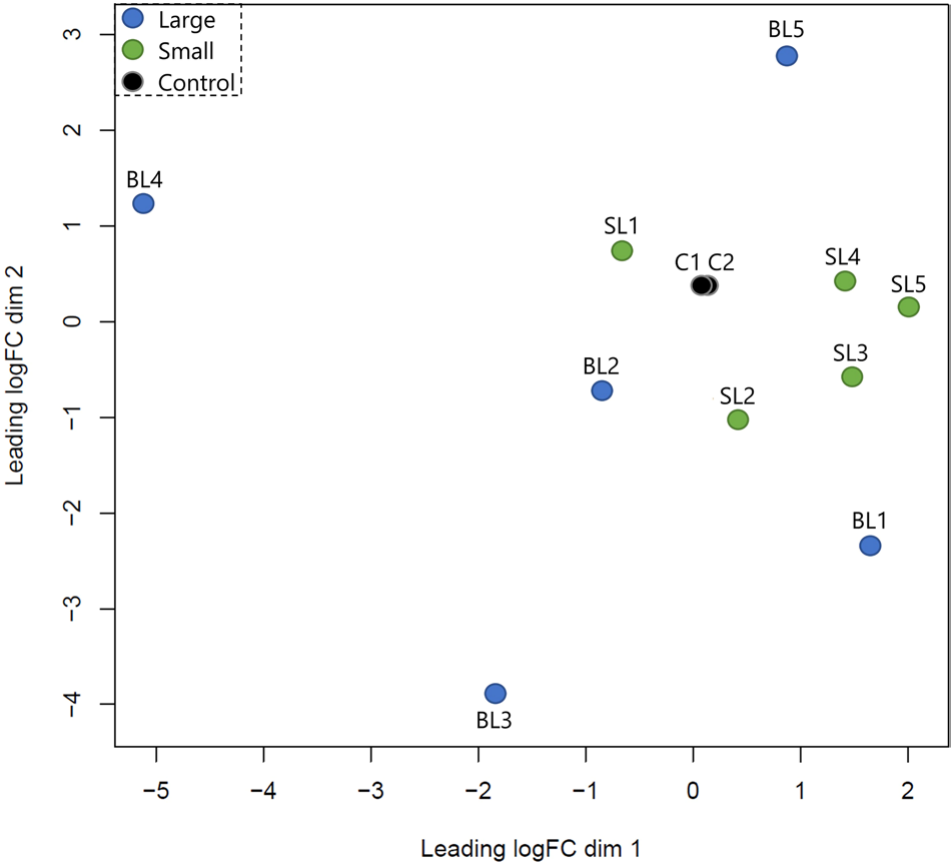
Multidimensional scaling plot of distances between RRHP libraries based on their log-transformed 5hmC counts. Black circles represent the control libraries, while blue and green represent the large- and small-sized individuals, respectively.

Aligned reads starting with CCGG in both strands were extracted in text files using a custom Python script (Supplementary File 5; please see code availability statement). RRHP produces count-based datasets and their analysis is similar to that of RNA-Seq count-based datasets for differential expression. Therefore, the output text files were used as input in R where they were filtered twice based on 5hmC counts. At first, 5hmC positions were eliminated when ≥ 6 of all samples from both groups had zero counts. This threshold was applied to remove sites with low 5hmC levels and at the same time ensure that eliminated sites had zero counts across both groups. Since thresholds can be subjective, for the second step of filtering we calculated the median of 5hmC counts across the entire dataset (Ymax = 19), and removed 5hmC sites when ≥ 6 samples had counts below the median (Supplementary File 6; please see code availability statement). Using limma software^29^ in R, we log2 transformed the count data using the function voom and a linear model was fitted (function lmFit) using weighted least squares which incorporate the variance of each observation into the regression. Variability estimates were then adjusted using the eBayes function. The log-transformed dataset including the control libraries was plotted in a multidimensional scaling plot (Fig. 3). Differentially hydroxymethylated sites between the two groups were extracted based on the adjusted p value (q < 0.05). The false discovery rate (FDR) was controlled using the Benjamini-Hochberg correction, which is the default in limma.

### DhmC annotation using HOMER

For the annotation of DhmCs, the annotatePeaks.pl perl script was used. This script is available upon installation of the HOMER software on LINUX systems (http://homer.ucsd.edu/homer/introduction/install.html). Since the Nile tilapia genome is not included in HOMER’s database, we performed a custom gene annotation as shown in the manual (http://homer.ucsd.edu/homer/ngs/annotation.html) and explained in detail within Supplementary File 6 (please see code availability statement). In brief, DhmC information from *toptable* output was adjusted to fulfil HOMER requirements. The resulting BED file had 6 columns separated by TABs. The first column is the chromosome information, second column - start position, third column - end position, forth column - unique peak ID (which can be digits from 1–2,677 counting the total number of DhmCs), fifth column can be empty or include any desired information to be retained after the annotation is completed (i.e. paste columns from *toptable* such as adjusted.p.values) and the sixth column - strand information (+/− or 0/1, where 0 = “+”, 1 = “-”).

### Interactive visualisation of the data

An Integrative Genomics Viewer^30^ (IGV) session (Related Manuscript Files 1–6) has been created for exploration of 5hmC positions across the Nile tilapia genome in individual samples and visualisation of DhmC results as bar plots or heatmaps. Detailed instructions are provided in Supplementary File 7 (to access the IGV session please see section “Data Records”).

## Data Records

The raw fastq files produced in this work are deposited in SRA under the accession number: SRP285050^31^ and consist of 2 controls (C1 and C2) derived from a single library sequenced in both flow cells, 5 liver samples (BL1-BL5) that represent the large-sized individuals and 5 liver samples (SL1-SL5) which represent the small-sized individuals (Table 2). Supplementary Files 1–4, as well as the IGV session (Supplementary File 7) for visualization of the RRHP dataset between large and small female Nile tilapia were deposited on DataverseNO^32^. The files can be found at the permanent DOI link: 10.18710/6VWVMQ.

**Table 2:** Experimental setup and output per sequenced library.

Group	SampleID	SRA Accession	Experimental manipulation	Raw Reads	Total 5hmCs	Unique 5hmCs	Across Samples			
							Unique 5hmCs	First filter	Second filter	DhmCs (B *vs* S)
Control	C1	SRS7585929	RRHP - Second MspI digestion omitted	45,994,060	22,618,039	611,908	1,096,820	405,605	138,000	2,677
	C1	SRS7585930	RRHP - Second MspI digestion omitted	34,339,125	16,324,747	556,289				
Large	BL1	SRS7412736	RRHP Protocol	29,951,172	4,702,129	411,812				
	BL2	SRS7412737	RRHP Protocol	64,951,163	15,790,930	517,979				
	BL3	RS7412738	RRHP Protocol	46,520,470	17,040,820	478,241				
	BL4	SRS7412739	RRHP Protocol	65,123,992	27,719,382	544,846				
	BL5	SRS7412740	RRHP Protocol	56,155,408	18,040,871	534,659				
Small	SL1	SRS7412741	RRHP Protocol	21,105,349	3,441,132	371,621				
	SL2	SRS7412742	RRHP Protocol	27,287,570	7,637,187	383,449				
	SL3	SRS7412743	RRHP Protocol	7,105,158	2,466,658	279,178				
	SL4	SRS7412744	RRHP Protocol	24,225,784	6,106,860	389,376				
	SL5	SRS7412745	RRHP Protocol	12,690,867	3,898,492	318,951				

Total 5hmCs represent aligned hydroxymethylated cytosines across the Nile tilapia genome, while DhmCs are differentially hydroxymethylated cytosines in liver between large- and small-sized individuals (p < 0.05). B represents the large- and S the small-sized group, respectively.

## Technical Validation

To avoid technical biases during library preparation, the samples were given random numbers prior to measuring DNA concentrations. The libraries were prepared simultaneously and the initial DNA concentration was the same for all samples. After final amplification and purification of all RRHP libraries, the groups were traced back and assigned to each sample. Differences in DNA hydroxymethylation levels across samples and groups were evident prior to sequencing as shown in Fig. 2a.

Overall, the sequencing quality of every library was high (average Phred Score > 32 per base pair) and it was measured using FastQC and MultiQC as shown in Fig. 2. Sequencing lane bias was tested by plotting the log-transformed count data among samples and positive controls in a multidimensional scaling plot (Fig. 3). The two controls were overlapping showing no significant differentiation; therefore, no action was needed regarding normalization of counts between flow cells.

## Data Availability

Supplementary files 5 and 6 were deposited in GitHub on 2022/12/20^33^. They can be found at the URL: https://github.com/IoannisKonstantinidis/RRHP_Code.
